# Immunoinformatics- and Bioinformatics-Assisted Computational Designing of a Novel Multiepitopes Vaccine Against Cancer-Causing Merkel Cell Polyomavirus

**DOI:** 10.3389/fmicb.2022.929669

**Published:** 2022-06-28

**Authors:** Nahlah Makki Almansour

**Affiliations:** Department of Biology, College of Science, University of Hafr Al Batin, Hafr Al Batin, Saudi Arabia

**Keywords:** Merkel cell polyomavirus, multi-epitopes vaccine, molecular docking simulation, binding free energies, immunoinformatics

## Abstract

Merkel cell polyomavirus (MCV) contains double-stranded DNA as its genome and is the fifth polyomavirus that infects humans. The virus causes Merkel cell carcinoma (aggressive skin cancer). Till present, no proper drug or vaccines are available to treat/prevent the virus infection and stop the emergence of Merkel cell carcinoma. In this study, computational vaccine design strategies were applied to design a chimeric-epitopes vaccine against the virus. The complete proteome comprised of four proteins was filtered through various vaccine candidacy parameters and as such two proteins, namely, capsid protein VP1 and capsid protein VP2, were considered as good vaccine targets. Furthermore, they harbor safe and potential B and T cell epitopes, which can be used in a chimeric multiepitopes-based vaccine design. The epitopes of the vaccine have maximum world population coverage of 95.04%. The designed vaccine structure was modeled in 3D that reported maximum residues in favored regions (95.7%) of the Ramachandran plot. The interactions analysis with different human immune receptors like TLR3, MHC-I, and MHC-II illustrated vaccine's good binding affinity and stable dynamics. The structural deviations of the vaccine receptor(s) complexes are within 5 Å, where majority of the receptors residues remain in good equilibrium in the simulation time. Also, the vaccine was found to form between 60 and 100 hydrogen bonds to receptors. The vaccine stimulated strong immune responses in addition to interferon and cytokines. The strength of vaccine-receptor(s) binding was further affirmed by binding energies estimation that concluded <-150.32 kcal/mol of net binding energy. All these findings suggest the vaccine as a promising candidate that needs further experimental testing to disclose its real immune protective efficacy. Furthermore, the designed vaccine might accelerate vaccine development against the MCV and could save time and expenses.

## Introduction

The polyomaviruses family consists of non-enveloped viruses that have a circular ds-DNA genome (Dalianis and Hirsch, 2013). The size of genome is ~5.4 kilo base pairs. The family is named because certain members carry the ability to induce malignancy in various types of experimentally infected organisms (Schowalter et al., 2010). The novel Merkel cell polyomavirus (MCV also known as MCPyV) is the fifth member of the polyomaviridae family of the murine polyomavirus group (Stakaityte et al., 2014). MCV has 5,387 base pairs circular genome that is considered as one of the known seven human tumor-causing viruses (Feng et al., 2008). MCV carries double-stranded DNA as its genetic material (Schowalter et al., 2010). The virus carries a large tumor cell oncoantigen and a small tumor cell oncoantigen along with proteins forming the virus capsid. The capsid proteins are encoded by genes such as VP-1, VP-2, and VP-3 (Schowalter and Buck, 2013). The virus undergoes several mutations that produce a large truncated T antigen that has Rb-binding motif but lacks helicase and DNA binding domains (Knepper et al., 2019). The MCV-miR-M1-5p is the microviral RNA that is 22 nucleotides long, which is expressed by the MCV that automatically regulates the expression of genes in the later phase of infection (Hasham et al., 2020). MCV shows a relative degree of homology with T cell antigens and viral proteins of African green monkey polyomavirus (Bhatia et al., 2011). This virus majorly infects humans and is associated with oncogenesis (Akram et al., 2017). The MCV is associated with the unusual cutaneous form of neuroendocrine cancer, Merkel cell carcinoma (MCC), in humans (Feng et al., 2008). The association of MCV with MCC has aroused the interest of researchers in this virus. MCV is mainly polyomavirus type that is one of the causative agents of malignancy (MCC) in humans (Schowalter et al., 2010). Merkel cells are also known as “touch cells” are present in mucosal tissues, hair follicles, and in the epidermis basal layer of skin (Halata et al., 2003; Moll et al., 2005). The mechanism of MCV life cycle includes; early T cell proteins which are immediately activated and involved in DNA transcription, whereas late proteins guide replication of structural components i.e., viral capsids and products used in progeny production (Arora et al., 2012; White et al., 2014).

The MCV was first isolated from MCC patient's tissue samples (Wong et al., 2015). Various researchers from the United States and Europe have confirmed the presence of 69–85% of MCV DNA in MCC neuroendocrine tumors (Houben et al., 2010). Due to the slow and steady incidence of the MCC, the paraffin-embedded formalin-fixed tissues analysis of MCC is performed to assess the prevalence of MCV (Mangana et al., 2010). MCV presence is reported in several anatomical locations, including the respiratory tract, saliva, gastrointestinal tract, urine, and lymph tissues, but its mechanism is very slow in these areas, whereas on dermal tissues, its mechanism of action is relatively high where transcription of both DNA and viral capsids is found (Andea et al., 2014). The etiological and biological transmission of virus is usually associated with asymptomatic primary infection in early childhood either *via* cutaneous, respiratory, or fecal-oral routes (Spurgeon and Lambert, 2013). The virus is usually the normal microflora of the skin, but due to mutation and integration in the host chromosome, i.e., in Merkel cells, induced suppression of immune cells by drugs that may occur in organ transplant along with the aging, AIDS, and ultraviolet radiations may transform the virus pathogenic (Liu et al., 2016). The development of painless, plaque-like tumor small outpost lesions on the skin may be diagnosed by blood tests, viral DNA detection by Southern blotting, and PCRs most commonly, whereas the viral infection is asymptomatic (Schadendorf et al., 2017).

The infection may be prevented or therapeutically treated to limit prolonged exposure to sun rays and avoid exposure to UV radiations and prevent genome replication of viral cells of the skin. No vaccines and medications are available at present time to prevent MCV infection. The primary treatment of the infected tumors is carried out by radiotherapy, i.e., adjuvant radiotherapy and local surgical excision of tumors in the early stages. Additionally, chemotherapy and surgical excision are important treatment options and checkpoint inhibitors show promising results and are part of the treatment in the clinics (Tai, 2013). Cancer immunotherapy includes checkpoint inhibitor treatment. Checkpoint inhibition is an effective treatment approach for treating MCC. Immune checkpoints are drugs that target immune cells' proteins called checkpoints. The checkpoints allow immune responses to either be strong or stop T cells from killing cancerous cells thus like switches to either turn “ON” or “OFF” immune responses (Franzin et al., 2020). Avelumab is currently used as a licensed checkpoint treatment for metastatic disease (Knepper et al., 2019). Vaccine development against MCV is a promising strategy to prevent virus infection (Zhang, 2018). Traditional vaccine is time consuming and very expensive (Delany et al., 2013). In this regard, computational vaccine designing could offer an attractive alternative platform to propose a novel vaccine construct for experimentalists to test the theoretical vaccine in tackling the pathogen (Seib et al., 2009; Albekairi et al., 2022a,b; Ud-din et al., 2022). The computational vaccine design work on genomic information and uses a set of filters based on experimental data to identify the most suitable antigenic epitopes for experimental testing (Adu-Bobie et al., 2003; Suleman et al., 2021; Alharbi et al., 2022a,b). Such techniques have been successfully used for the development of vaccines against several bacterial pathogens (Pizza et al., 2000; Naz et al., 2021; Ullah et al., 2021; Fatima et al., 2022). In this work, subtractive proteomics and reverse vaccinology filters were used to identify suitable vaccine antigenic epitopes, which were then used in a chimeric vaccine design MCV. Thus, we believe that findings of this study might be useful for experimentalists in designing a successful vaccine against MCV.

## Materials and Methods

**Figure 1:**
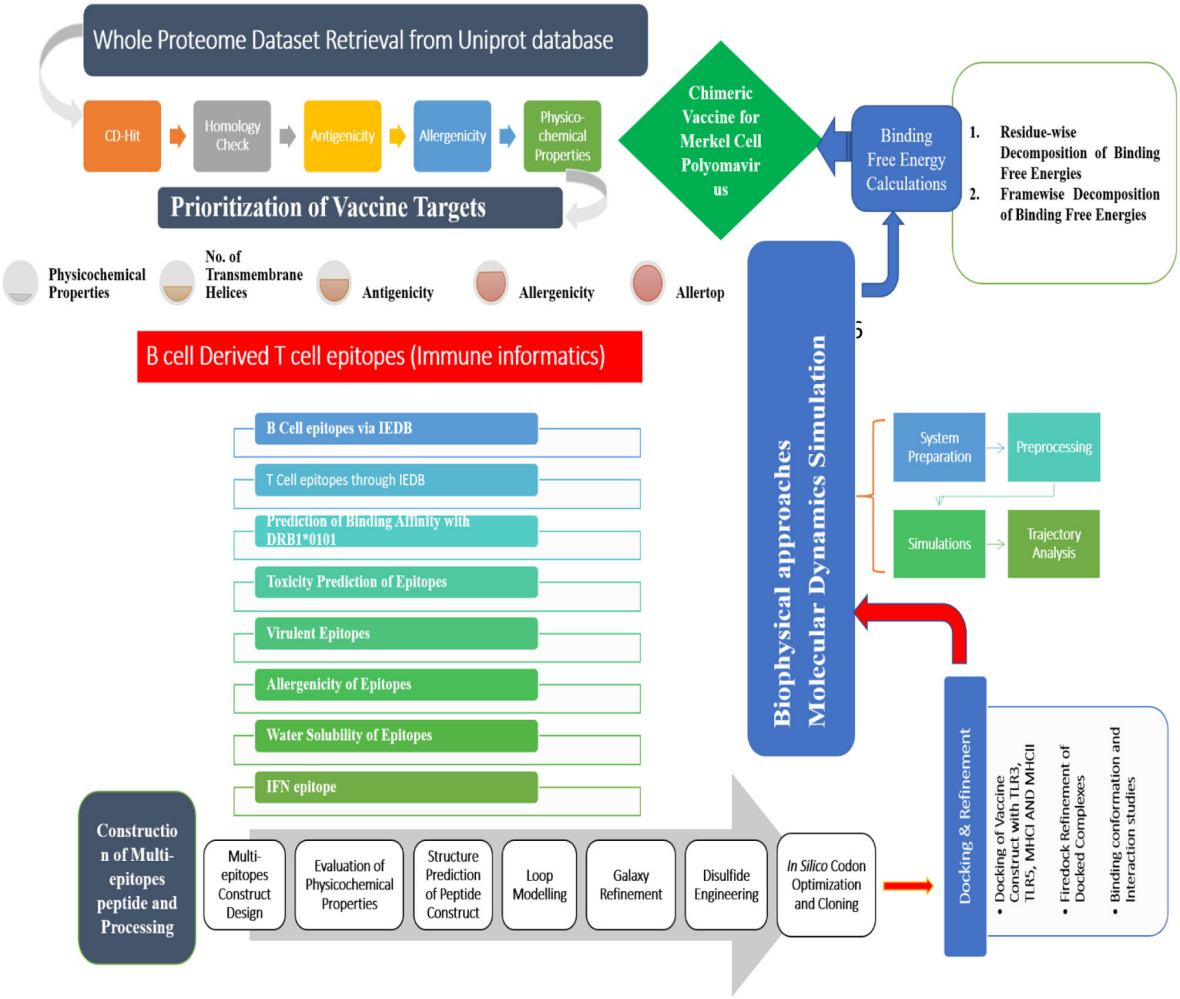
A complete step-by-step methodology followed in this study. The study is started with the retrieval of complete protein dataset of the virus and subjected to several immunoinformatic filters to identify potential antigenic epitopes. This was followed by multiepitopes vaccine construction, molecular docking, and molecular dynamics simulation analysis.

The different methods used in this study for developing a vaccine against MCP are schematically described in Figure 1.

### Prioritizing MCV Vaccine Candidates

The proteins dataset of MCV was retrieved from the UniProt Proteome database (Consortium, 2014) and analyzed for prospective vaccine candidates. First of all, the proteome was subjected to BLASTp to remove human homologous proteins. The proteins showing homology with the human host (taxonomic id: 9606) were discarded using an online BLASTp tool (Blast, 2015). Proteins with an E-score of 1.0 E^−5^, a bit score of more than 100, and a sequence identity of >30% were not chosen and characterized as host homologs. TMHMM 2.0 (Chen et al., 2003) was used to calculate transmembrane helices of the screened host non-homologous proteins and those with transmembrane helices number <than 1 were selected, and protein sequences having >1 transmembrane were discarded. Less number of transmembrane helices ensures easy cloning and expression of the proteins (Ismail et al., 2021). The filtered proteins were further investigated for allergenicity, antigenicity, and physicochemical properties using AllerTOP 2.0 (Dimitrov et al., 2014), VaxiJen 2.0 (cutoff ≥ 0.4) (Doytchinova and Flower, 2007), and ExPASY Protparam (ProtParam, 2017), respectively.

### Mapping of B- and T-Cell Epitopes

The immune epitope database (IEDB) server (Vita et al., 2018) was utilized to predict B- and T-cell epitopes. Bepipred Linear Epitope Prediction 2.0 (Jespersen et al., 2017) was first used to predict linear B-cell epitope considering a cutoff (0.5), which were subsequently subjected to T-cell epitope mapping to forecast subsequences that have good binding potential to reference set of MHC alleles. For each epitope, the percentile score was determined, and only low percentile score epitopes were categorized as efficient binders. Following that, the chosen epitopes were used in MHCPred 2.0 (Guan et al., 2003) to shed light on the epitopes DRB^*^0101 allele binding potential (cut-off value, 100 nM). ToxinPred (Gupta et al., 2015) and VaxiJen 2.0 (Doytchinova and Flower, 2007) online tools were used further to affirm the selection of nontoxic and antigenic epitopes, respectively. Allergenic epitopes were discarded if found positive in AllerTOP 2.0 (Dimitrov et al., 2014). An Innovagen tool (http://www.innovagen.com) was used to evaluate epitopes' water solubility potential. The IFNepitope server (Dhanda et al., 2013) was used to predict inferon-gamma producing epitopes. The population coverage analysis of the final set of epitopes was carried out using the IEDB population coverage analysis tool (http://tools.iedb.org/population/).

### Multiepitopes Vaccine Designing

The eligible immune-dominant epitopes were fused to design a multiepitope peptide vaccine (Abdelmoneim et al., 2020; Ismail et al., 2020b; Tahir ul Qamar et al., 2021). A major problem in peptide vaccines is their low immunogenicity that can be overcome by a multiepitope peptide with a suitable adjuvant molecule (Li et al., 2014; Ahmad et al., 2019; Gul et al., 2022). The vaccine built in this investigation used AAY linkers to join the filtered epitopes. Beta-defensin was added to the N-terminal of vaccine as an adjuvant (Ferris et al., 2013) to boost the immunogenicity of the designed vaccine construct. The vaccine tertiary structure was generated using the 3Dpro software (Cheng et al., 2005) to understand its binding with host immune cell receptors in docking and simulation analysis. GalaxyLoop (Giardine et al., 2005) and GalaxyRefine (Heo et al., 2013) were used further to model the loops and improve the vaccine structure by removing structural errors.

### Vaccine Physicochemical Properties and Host Immune Simulation

To facilitate experimental studies, the ExPASY ProtParam (ProtParam, 2017) was used to determine physicochemical properties of designed vaccine. The instability index is a critical parameter as it helps in the elimination of unstable peptide candidates. The designed vaccine was then evaluated for its potential of stimulating the types of host immune responses using the C-ImmSim server (Rapin et al., 2012).

### Docking and Refinement

Molecular docking was applied to predict the conformation of vaccine different innate immune receptors, including TLR3 (PDB ID: 2A0Z), MHC-I (PDB ID: 1I1Y), and MHC-II (PDB ID: 1KG0) (Sussman et al., 1998). Blind docking was performed through an online PATCHDOCK (Schneidman-Duhovny et al., 2005). Cluspro and GRAMMX are FFT-based methods, while PATCHDOCK works on shape complementary principle. Molecular docking with PATCHDOCK results in small steric clashes, and the findings are more reliable as they are cross-validated by FireDock (Andrusier et al., 2007). The complexes were visualized for binding using the UCSF Chimera 1.13.1 software (Kaliappan and Bombay, 2016). The complex with lowest global binding energy was chosen for investigation of intermolecular interactions.

### Molecular Dynamics Simulations

To get insight into how the vaccine behaves dynamically with the receptors, molecular dynamics simulations were performed. The simulation study was also critical for validating the epitopes' exposure to the human immune system. The antechamber module of AMBER20 (Case et al., 2020) was used to create the TLR3, MHCI, MHCII, and vaccine construct libraries. Solvation of the complexes was carried out in TIP3P solvation box (size 12 angstrom). Ff14SB (Maier et al., 2015) was used as force field, and systems were neutralized. The complexes' energy was accomplished by the steepest descent algorithm (1,000 cycles) and conjugate gradient algorithm (1,000 cycles). The systems were then heated to 300 K. Langevin dynamics (Izaguirre et al., 2001) was used to maintain the system's temperature while the SHAKE algorithm (Kräutler et al., 2001) was used to constrain hydrogen bonds. The systems were equilibrated and simulated for 300 ns. The CPPTRAJ module (Roe and Cheatham, 2013) was used to examine systems stability vs. simulation time.

### Estimation of Binding Free Energies

The MMPBSA.py module of AMBER20 was used to estimate the MMPBSA binding free energies (Miller et al., 2012; Genheden and Ryde, 2015). Using the anteMMPBSA.py module, the initial files for the complexes, receptors, and vaccines were generated. In the analysis, 1,000 frames were evaluated from simulated trajectories (Ahmad et al., 2017).

### Disulfide Engineering and Vaccine Cloning

To stabilize the vaccine structure, disulfide bonds were added to the vaccine using Design 2.0 (Craig and Dombkowski, 2013). The vaccine was then reverse translated and optimized according to *Escherichia coli* codon usage using the JCat tool (Grote et al., 2005) to increase the expression of cloned vaccine sequence. The expression of cloned vaccine was evaluated using the GC content and codon adaptation index (CAI). The optimum CAI value considered is 1, and GC content range is between 30 and 70% (Ismail et al., 2020b). Cloning of the vaccine into the pET28a (+) vector was carried out through SnapGene.

## Results and Discussion

### Retrieval of MCV Proteins

The purpose of this study was to prioritize antigenic epitopes and to build a chimeric peptide vaccine against MCV using complete proteins dataset of the virus available in the Uniprot database. Numerous bioinformatics and immunoinformatics tools were used to aid experimentalists in the development of vaccines.

### Identification of MCV Vaccine Targets

Identification of good vaccine candidates may aid in reducing the amount of time, effort, and resources required to develop and optimize an active vaccine against a given disease (Aslam et al., 2021; Ismail et al., 2021; Qamar et al., 2021). A total of 4 proteins (i.e., POLY small T antigen V7, POLY capsid protein VP1, POLY minor capsid protein VP2, and POLY large T antigen) were extracted and evaluated for sequence homology against human proteome. This was important to disclose as sequence homology between the viral protein(s) and the host may result in severe autoimmune reactions (Baseer et al., 2017). All 4 proteins were found non-homologous against the host proteome. Human vaccinations are often studied experimentally in mice due to the many practical benefits they provide. To guarantee the selection of non-similar mouse proteins, a homology check was added to the pipeline. This examination resulted in the identification of tr|B0G0W3|B0G0W3_9 POLY capsid protein VP1 and tr|B0G0W4|B0G0W4_9POLY minor capsid protein VP2 as mouse non-similar proteins. The non-identical proteins in mice will assist in avoiding false-positive findings during *in vivo* experiments and inaccurately interpreting the immune protection efficacy of selected vaccine candidates against the virus. Following that, transmembrane helices were counted in 2 shortlisted proteins. This transmembrane topological analysis is thought critical as such low number transmembrane helices allowed easy purification of proteins during vaccine development. Both screened proteins were observed to have no transmembrane helices. Antigenicity measures protein's binding capacity to antibodies or T-cells. The proteins were identified as antigenic and scored higher than a threshold (>0.4). Similarly, both proteins were found to be non-allergic, making them potential protein candidates for epitope prediction. Physicochemical properties of the shortlisted proteins were calculated *via* ExPASY protparam web tool and are tabulated in Table 1.

**Table 1:** Physicochemical properties of virus proteins.

**Proteins**	**POLY small T antigen V7**	**POLY capsid protein VP1**	**POLY minor capsid protein VP2**	**9POLY large T antigen**
BLASTp (Human)	No significant similarity found	No significant similarity found	No significant similarity found	No significant similarity found
Mus musculus-coverage/Identity	86%	No similarity	No similarity	97%
	36.65%	No similarity	No similarity	41.18%
Bit score	101	No similarity	No similarity	568
TMHMM	ND	0	0	ND
HMMTOP	ND	Chance	NOT	ND
Allergenicity	ND	Non-Allergen	Non-Allergen	ND
Vaxigen	ND	0.4374 (Probable ANTIGEN)	0.6649 (Probable ANTIGEN)	ND
Length	ND	423	241	ND
Molecular weight	ND	46.56	25.79	ND
PI	ND	8.25	4.66	ND
Aliphatic index	ND	78.72	124.61	ND
Instability	ND	37.87	46.08	ND
Gravy	ND	−0.425	0.379	ND

*ND, not done*.

### Epitopes Prediction

**Figure 2:**
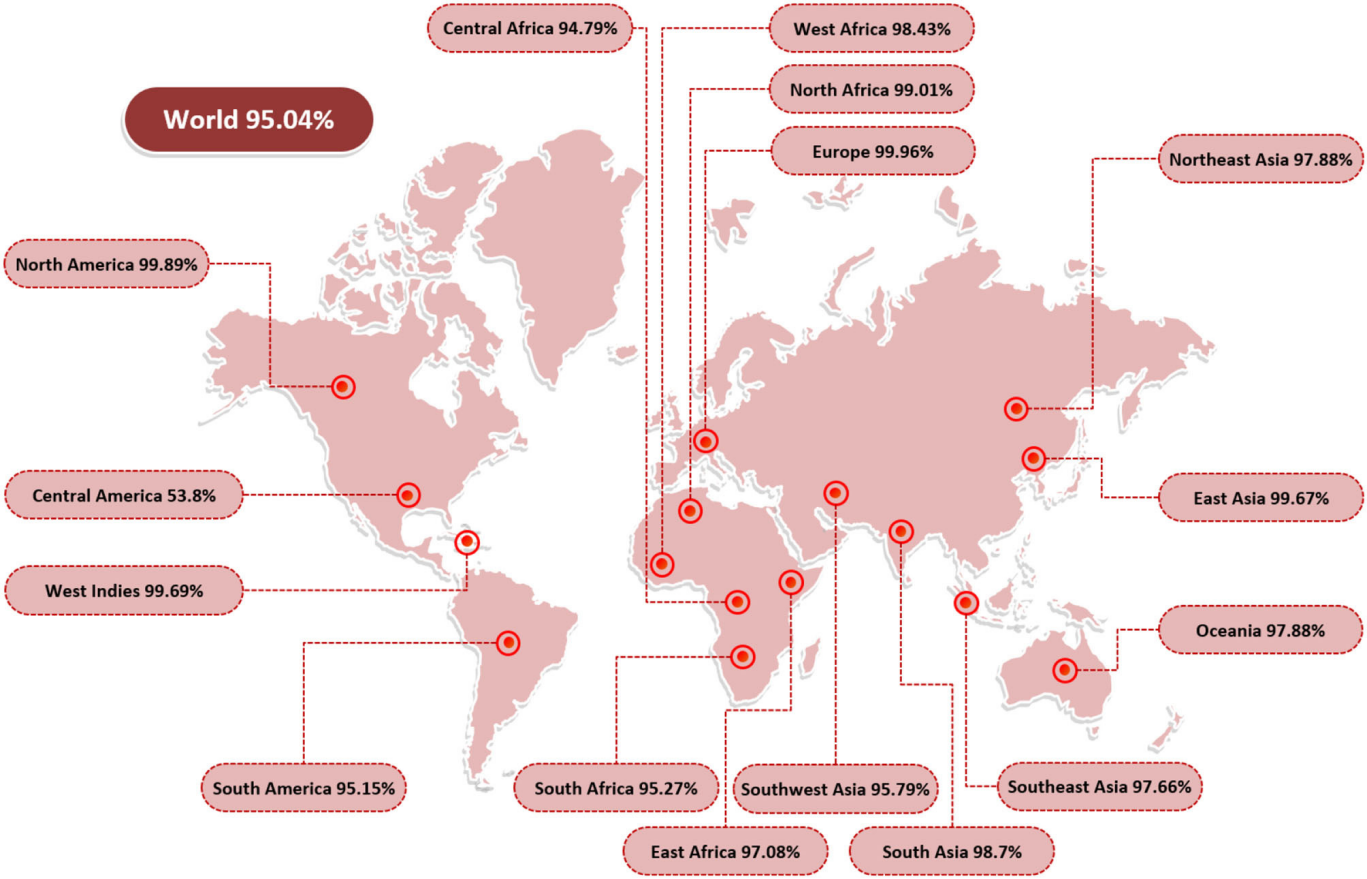
World population coverage of the final set of epitopes. The population coverage analysis was carried out using the IEDB population coverage tool and demonstrated the world population coverage by the set of selected epitopes.

Epitope identification aids in understanding disease origin, developing diagnostic tests, and epitope-based vaccinations. The adaptive immunity is highly specific and capable of recognizing and eliminating the invading pathogens. Additionally, adaptive immunity is capable of remembering antigen on successive encounters and thus forms a lifelong protective memory. B and T cells are key parts of the host adaptive immunity. Antigens bind to and activate particular receptors of the B and T cells. The vaccine candidates selected were subjected to B-cell epitopes prediction. For each protein, linear B-cell epitopes were predicted; eight epitopes were predicted for capsid protein VP1 and five for VP2. These B-cell epitopes are identified by B-cell receptors (BCRs) and upon activation release antibodies to neutralize antigen. Additionally, the proteins were examined for T-cell epitopes using a rigorous *p*-value cutoff of <0.005. The epitopes varied in length and interacted with a variety of MHC-I and MHC-II alleles. Capsid protein VP1 was found to have 48 T-cell epitopes, while capsid protein VP2 was predicted to contain 29 T-cell epitopes. MHC-I epitopes are recognized by CD8 (cytotoxic T lymphocytes), while MHC II epitopes are identified by CD4 T cells. Later on, CD4 T cells differentiate into helper T cells to boost immune responses against the infectious agent. A total of 7 epitopes for capsid protein VP1 (i.e., KRKASSTCK, RVHDYGAGI, ITIETVLGR, KMTPKNQGL, MPKVSGQPM, FGQEKTVYP, and KASQKESQT) and 3 epitopes for capsid protein VP2 (i.e., TIEGISGIE, VSLVKRDVS, and GTLQQQTPD) were shortlisted. These epitopes fulfilled all the parameters used in the study including antigenicity, allergenicity, toxicity, water solubility, and IFN-gamma production. Table 2 summarizes the final chosen epitopes that passed all of these criteria. The world population coverage of selected epitopes is 95.0% (Figure 2).

**Table 2:** Final set of epitopes predicted from potential two vaccine candidates (capsid protein VP1 and capsid protein VP2).

**Proteins**	**Final epitopes**	**MHC-Pred**	**Allergenicity**	**Antigenicity**	**Toxicity**	**Water solubility**	**IFN epitope**
Capsid protein VP1	KRKASSTCK	48.42	Non-Allergen	1.1991	Non-Toxin	Soluble	Positive
	RVHDYGAGI	81.28	Non-Allergen	1.14	Non-Toxin	Soluble	Positive
	ITIETVLGR	21.13	Non-Allergen	0.4	Non-Toxin	Soluble	Positive
	KMTPKNQGL	45.08	Non-Allergen	1.7	Non-Toxin	Soluble	Positive
	MPKVSGQPM	81.47	Non-Allergen	0.7	Non-Toxin	Soluble	Positive
	FGQEKTVYP	8.81	Non-Allergen	0.4	Non-Toxin	Soluble	Positive
	KASQKESQT	39.99	Non-Allergen	0.6	Non-Toxin	Soluble	Positive
Capsid protein VP2	TIEGISGIE	8.2	Non-Allergen	1	Non-Toxin	Soluble	Positive
	VSLVKRDVS	82.72	Non-Allergen	0.8	Non-Toxin	Soluble	Positive
	GTLQQQTPD	100.46	Non-Allergen	0.6925	Non-Toxin	Soluble	Positive

### Multiepitope Vaccine Construction

**Figure 3:**
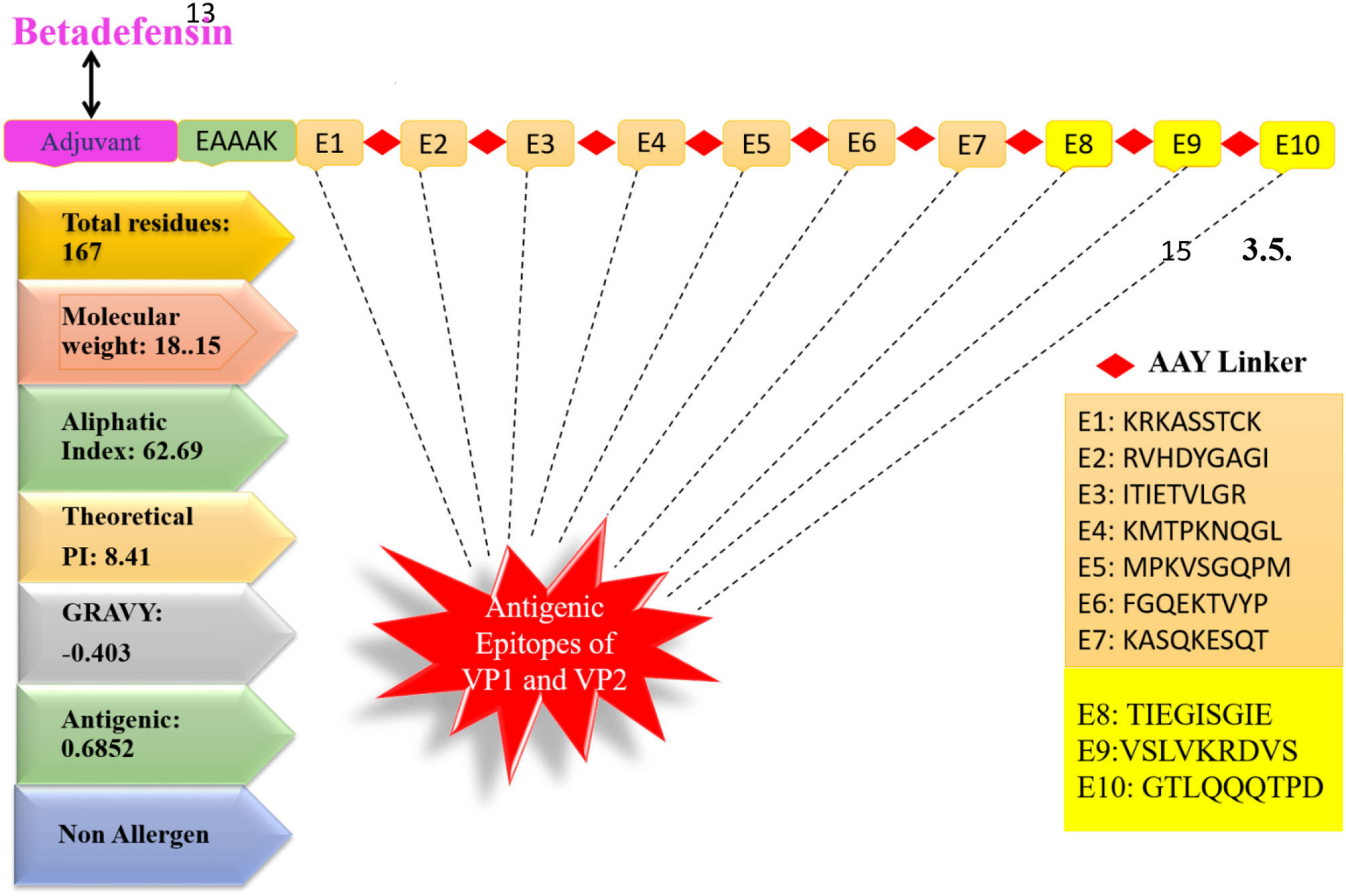
Schematic design of vaccine.

Multiepitope-based vaccinations are widely regarded as a good strategy for preventing and treating viral infections (Zhang, 2018; Ismail et al., 2020a). The epitopes indicated in Table 1 were fused using flexible AAY linkers, which enable the efficient separation necessary for each epitope to function well. After designing, an adjuvant of beta-defensin was added to its N-terminus of vaccine *via* the EAAAK linker. The linkers used allow efficient separation of the epitopes and will not allow them to fold on one another. Figure 3 illustrates the vaccine construct design schematically. Beta-defensin is a popular adjuvant due to its capacity of self-replication in a range of species and its ability to be linked to antigens by several chemical and genetic fusion techniques (Vemula et al., 2013). The designed vaccine was analyzed in the 3D structure analysis to predict the most suitable vaccine structure.

### Vaccine Physicochemical Properties

The vaccine sequence was used to determine different vaccine physicochemical properties (Figure 3). The vaccine design is 167 amino acids in length and has 2,539 atoms in total. The vaccine molecular weight is 18.15 kDa, thus it is easy to clone and express the vaccine in expression system (Baseer et al., 2017). The vaccine aliphatic index is 62.69, indicating a high degree of thermostability. A high aliphatic index value specifies the thermostability of the vaccine. The GRAVY score is −0.403 representing vaccine hydrophilic character. The GRAVY value ranges −2 to +2. The negative value demonstrates vaccine hydrophilicity. The predicted pI value of 9.52 indicates a somewhat acidic nature of the vaccine. However, to make the vaccine compatible to be administered into humans, a suitable buffer would be required to make its pH neutral.

### Vaccine Secondary and Tertiary Structure

**Figure 4:**
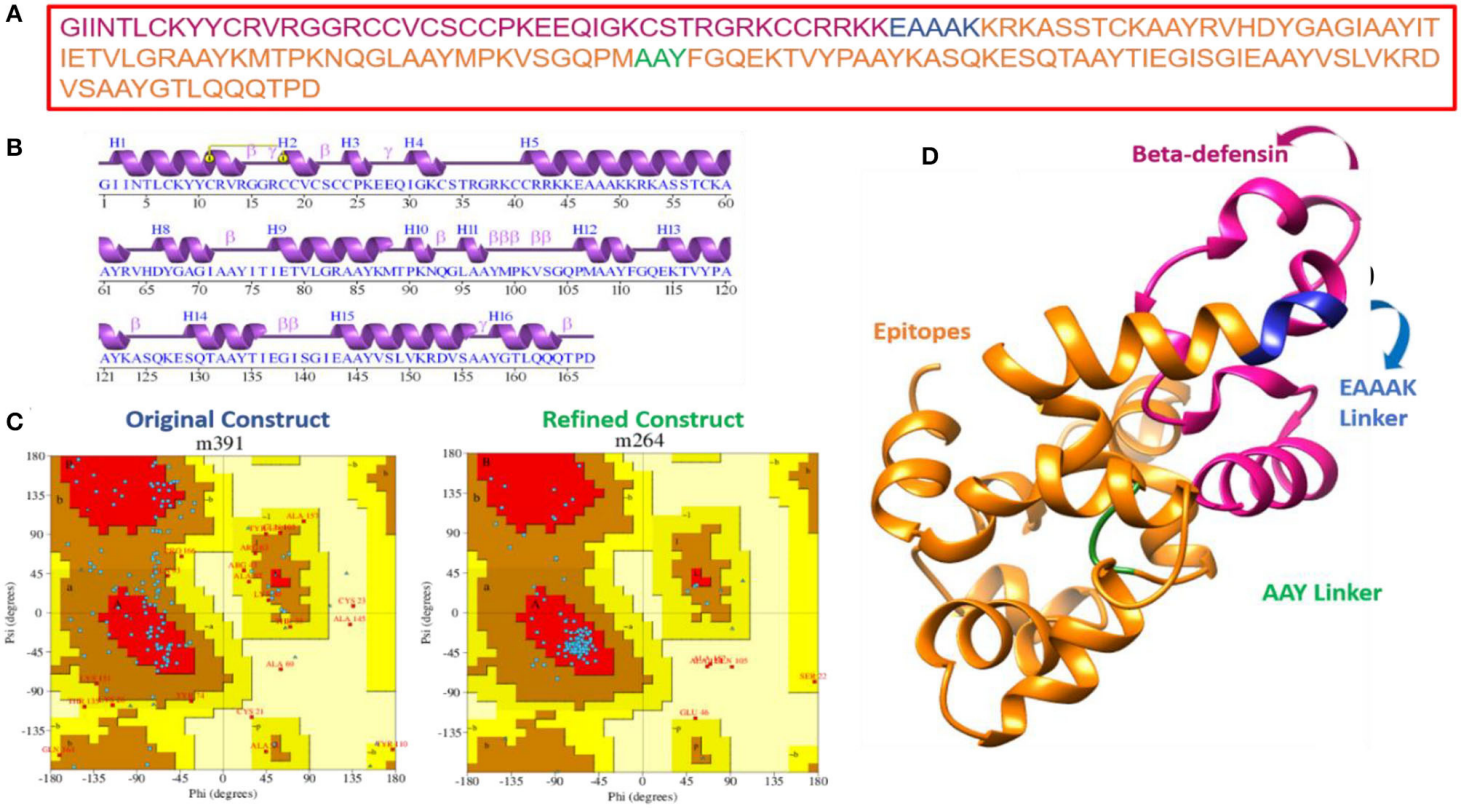
Primary, secondary, and tertiary structure analysis of the designed vaccine molecule. **(A)** Primary sequence of the vaccine, **(B)** vaccine secondary structure elements, and **(C)** Ramachandran plot for unrefined and refined vaccine models. The coloring pattern can be interpreted as; distribution residues across the core (denoted in red), allowed (denoted in brown), generously allowed (denoted in dark yellow), and disallowed (denoted in pale yellow) regions, and **(D)** 3D structure of refined vaccine model.

The primary sequence of the vaccine is presented in Figure 4A. The secondary structure components of the vaccine are as follows: 4 alpha helices, 51 beta-turns, 17 gamma turns, and 1 disulfide bond (Figure 4B). The original vaccine 3D structure was used in loop modeling where amino acid sequences, namely, Cys21, Cys24-Lys26, Gln29-Thr35, Cys41-Arg63, Asp66-Ile71, Ile77-Val80, Gly82-Met88, Pro90-Asn92, Met99-Lys101, Pro106-Phe111, Glu114-Lys123, Lys127-Ile136, Glu143-Lys151, and Tyr158-Gln164, were successfully modeled. Afterward, GalaxyRefine was employed to improve the overall vaccine structure quality. The model 1 was opted as an improved structure as it has a root mean square deviation (RMSD) value of 0.664 and a molprobity score of 1.399, which is relatively low in comparison to the initial structure score of 2.847, indicating that the modeled structure is of high quality. Similarly, the collision score is 18.1 times lower in comparison with the original structure, indicating that the structure lacks steric clashes. The vaccine net galaxy energy is very constant (−3,563.252), and the Ramachandran plot of favored residues improve from 87.3 to 90.9%. Table 3 summarizes the top ten refined models of the vaccine. The Ramachandran plot of unrefined vaccine comprises 87.3% of residues in the most favored regions, 42.5% in allowed, 11% in additionally allowed, and 2.7% in disallowed regions (Figure 4C). Figure 4D illustrates the vaccine 3D model structure. In contrast, for refined vaccine model, the Ramachandran plot revealed that 90.9% of the vaccine residues are in the most favored regions.

**Table 3:** Top-10 refined vaccine models.

**Model**	**RMSD**	**MolProbity**	**Clash score**	**Poor rotamers**	**Rama favored**	**Galaxy energy**
Initial	0	2.847	19.5	3.9	87.3	−2,143.69
MODEL 1	0.664	1.399	1.4	0	90.9	−3,587.34
MODEL 2	0.482	1.215	1.1	0	93.9	−3,570.72
MODEL 3	0.45	1.215	1.1	0	93.9	−3,570.54
MODEL 4	0.57	1.359	2.1	0	94.5	−3,563.42
MODEL 5	0.711	1.444	2.1	0	92.7	−3,558.33
MODEL 6	0.488	1.215	1.1	0	93.9	−3,557.64
MODEL 7	0.547	1.417	1.8	0	92.1	−3,557.05
MODEL 8	0.551	1.335	1.4	0	92.7	−3,556.94
MODEL 9	0.538	1.489	2.1	0	91.5	−3,555.8
MODEL 10	0.701	1.243	1.1	0	93.3	−3,554.74

*Statistics of the initial vaccine structure are also provided*.

### Vaccine Interactions With Immune Receptors

**Figure 5:**
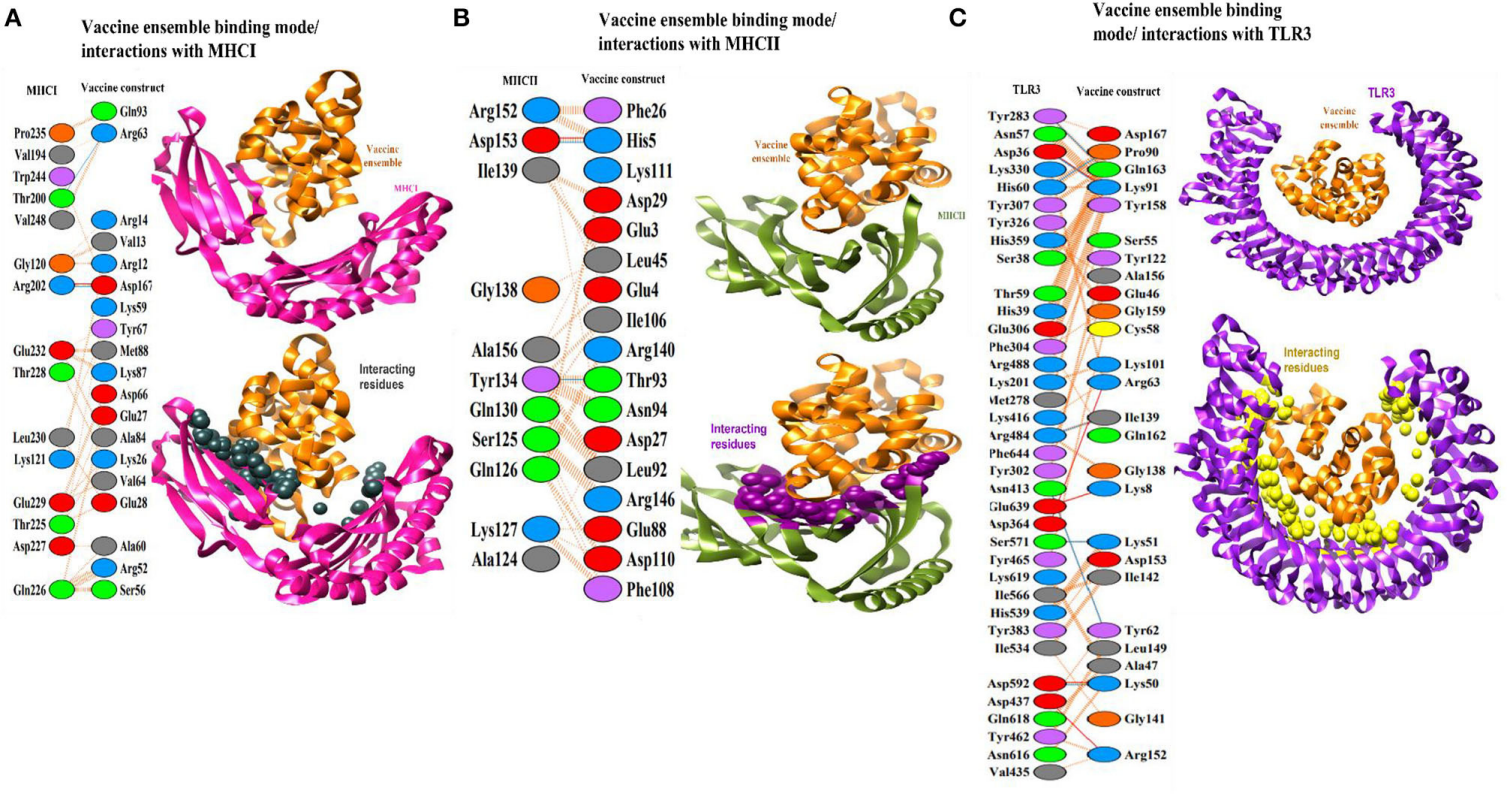
**(A)** Binding mode of vaccine (shown in organe cartoon) with TLR3 (shown in cartoon). The interacting residues are within 5 Å and are presented by yellow balls. **(B)** Binding mode of vaccine (shown in orange cartoon) with MHC-I (shown in magenta cartoon). The interacting residues are within 5 Å and presented by black balls. **(C)** Binding mode of vaccine (shown in orange cartoon) with MHC-II (shown in dark green cartoon). The interacting residues are within 5 Å and presented by maroon balls.

A protein-peptide docking technique was used to decode vaccine binding with TLR3, MHC-I, and MHC-II innate immune receptors. TLR3 triggers intracellular signaling pathways through the NF-B and stimulates production of inflammatory cytokines necessary for establishment of effective innate immunity (Matsumoto et al., 2011). These receptors identify molecular patterns associated with viruses and promote the production of interferons, therefore activating robust host defensive responses. Additionally, since adaptive immunity against antigens takes time to develop, it is critical to assess vaccine binding affinity for innate immune receptors (Ismail et al., 2020a,b). In each docking case, patchdock predicted ten docked solutions and ranked those according to the docking score (Supplementary Table 1). A high score indicates that the interacting molecules have a high affinity for one another and that the molecules have the best docked conformations with regard to one another. The best docked conformations and intermolecular interactions were observed in solution 7, 5, and 2 for TLR3, MHC-I, and MHC-II, respectively. FireDock refinement predicted solution 7 in case of TLR3 as best solution as it has a lower global binding energy of −23.22 kJ/mol (Supplementary Table 2). The attractive van der Waals energy contributes −20.40 kJ/mol to the net docking score, followed by the repulsive energy (8.70 kJ/mol), hydrogen bond energy (−4.81 kJ/mol), and atomic contact energy (ACE) of 4.08 kJ/mol. Within 5 Å, the vaccine was observed to interact with the following residues of TLR3 receptor: Tyr30, Asp36, Cys37, Ser38, His39, Asn57, Thr59, His60, Phe84, Val103, Glu127, Glu175, Leu177, Lys201, Met278, Tyr283, Tyr302, Phe304, Glu306, Tyr307, Tyr326, Asn328, His 359, Asp364, Tyr383, Asn413, Thr415, Lys416, Val435, Asp437, Leu440, Tyr462, Tyr465, Arg484, Arg488, Arg489, Ile510, Ile 534, His539, Ile566 Glu570, Ser571, Asp592, Leu595, Asn616, Gln618, Lys619, Glu639, Asp641, Arg643, Phe644, Asn667, and His674 (Figure 5A). Likewise, with a total global energy of −31.35 kJ/mol, attractive van der Waals energy of −26.69 kJ/mol, repulsive van der Waals energy of 8.72 kJ/mol, hydrogen bond energy of −1.84 kJ/mol, and ACE 12.17, solution 5 in case of vaccine docking with MHC-I was selected. The different docking parameters value of vaccine with MHC-I is shown in Supplementary Table 2. Visual inspection of the complex can be seen in Figure 5B. The following residues of MHC-I showed hydrogen bond and hydrophobic interactions with vaccine: Ile23, Tyr27, Gln115, Asp119, Gly120, Lys121, Asp122, Val194, Glu198, Thr200, Arg202, Thr214, Leu215, Thr216, Thr225, Gln226, Asp 227, Thr228, Glu229 Leu230, Val231, Glu232, Arg234, Pro235, Trp244, and Val248. In case of MHC-II, solution 2 has a lower global binding energy of −7.91 kJ/mol than the other anticipated solutions. The attractive van der Waals energy provides −17.34 kJ/mol to the total score, while the repulsive van der Waals energy is 10.6 kJ/mol, hydrogen bond energy is −41.77 kJ/mol, and atomic contact energy is 3.54 kJ/mol. Dock pose of vaccine-MHC-II is illustrated in Figure 5C. The MHC-II residues that showed interactions with vaccine within 5 Å are Glu3, Glu4, His5, Phe26, Asp27, Gly28, Asp29, Arg44, Leu45, Glu47, Glu88, Thr90, Val91, Leu92, Thr93, Asn94, Ser95, Ile106, Phe108, Asp110, Lys111, Pro139, Arg140, Arg146, and Phe148. According to docking studies, the vaccine demonstrated robust interactions with human innate immune receptors.

### Host Immune System Simulation

**Figure 6:**
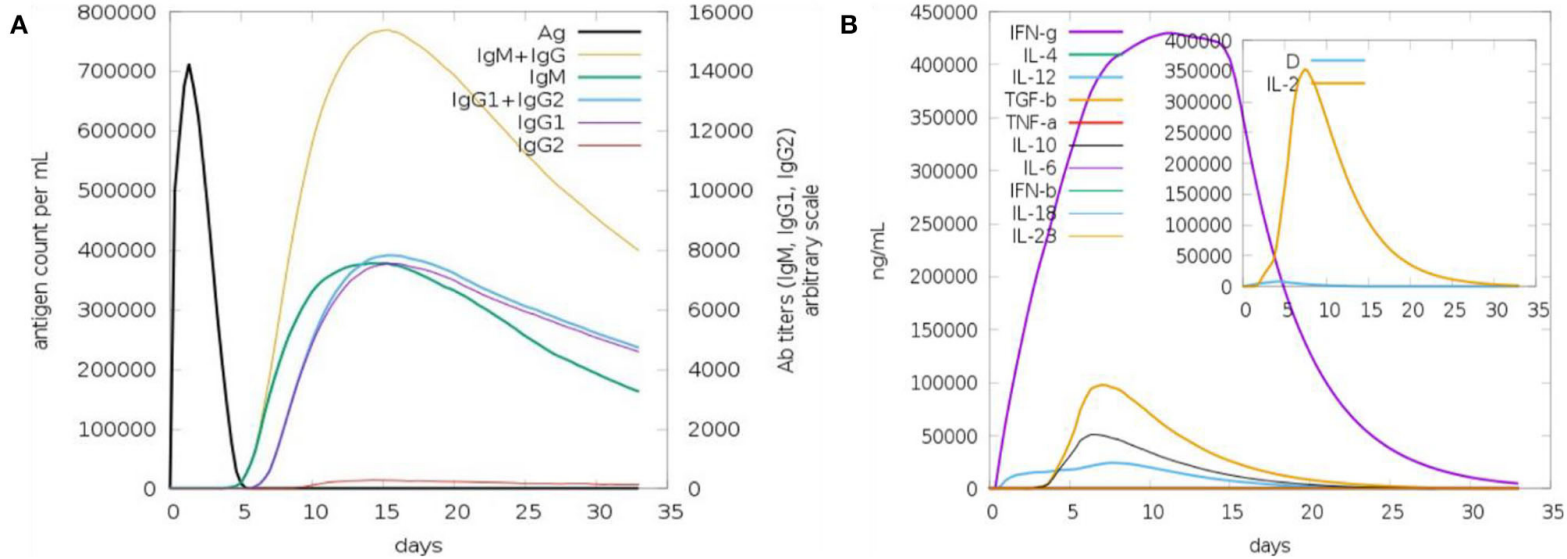
Host immune response to the vaccine antigen. **(A)** Antibodies titer vs. days, **(B)** interferon and cytokines production in response to the vaccine antigen.

Upon injection of the vaccine antigen into the host body, the vaccine elicited substantial immune responses (Figure 6). As can be seen that IgM and IgG antibodies have high titer (7,000 antigen count/ml), followed by IgM antibodies (>3,000 antibody titer per ml). The combination of IgG1 and IgG2 and IgG1 produced high antibodies titer. The IgG2 antibody response is modest. The interferon gamma (IFN-γ) generated in response to the antigen is more than 400,000 ng/ml. Additionally, other cytokines and interferon were reported to play a contribution in clearing the pathogen.

### Molecular Dynamics Simulation

**Figure 7:**
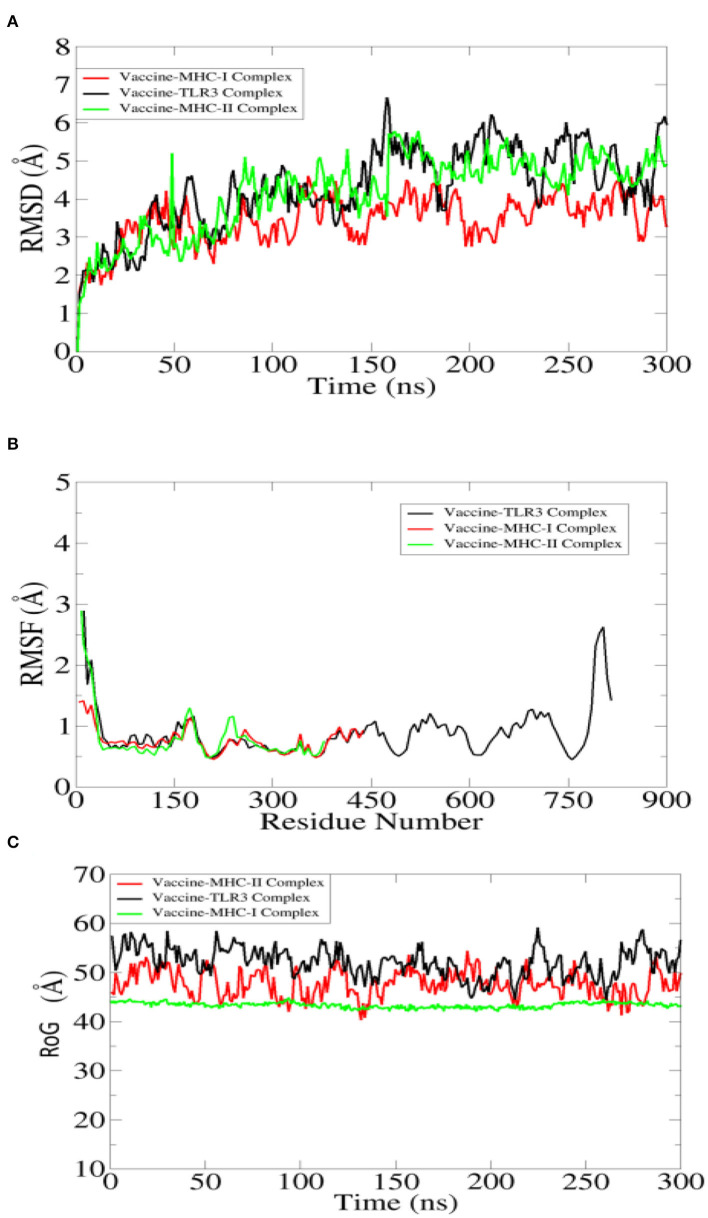
Statistical analysis of simulation trajectories. **(A)** Root mean square deviation (RMSD), **(B)** root mean square fluctuation (RMSF), and **(C)** radius of gyration (RoG).

The designed vaccine dynamics with TLR3, MHC-I, and MHC-II vs. time were deciphered through molecular dynamics simulation of 300 ns. The simulation trajectories were analyzed using root mean square deviation (RMSD) (Kuzmanic and Zagrovic, 2010; Altharawi et al., 2021), root mean square fluctuation (RMSF) (Ahmad et al., 2017; Qamar et al., 2021), and radius of gyration (RoG) (Lobanov et al., 2008; Ehsan et al., 2018) assays as depicted in Figure 7. For the TLR3-vaccine complex, the net RMSD of 4.30 Å while for MHC-I vaccine and MHC-II vaccine had an average RMSD of 3.45 Å and 4.59 Å, respectively (Figure 7A). During simulation, receptor molecules (i.e., TLR3, MHC-I, and MHC-II) were seen as more compact than vaccine, and as a consequence, free movement of the vaccine was reported though it docked quite stable with the receptors. The second statistical metric calculated for complexes was RMSF, which represents dynamical residue fluctuations over a certain time period. The mean RMSF determined for the TLR3-vaccine system is 1.5 Å, while for MHC-I vaccine and MHC-II vaccine, the mean RMSF is 0.7 Å and 0.9 Å, respectively (Figure 7B). A majority of receptor residues exhibit less variability and are quite stable. Higher RMSF values correspond to residues that are present in either receptor or vaccine loops and are dynamically more flexible than other secondary structure elements residues. The RoG study confirmed the receptors' stability in all complexes, revealing a stable plot with a mean value of 55.80 Å for TLR3 vaccine, 45.01 Å for MHC-I vaccine, and 49 Å for MHC-II vaccine complex (Figure 7C). The RoG results are consistent with those of RMSD in terms of interpreting systems' stability and the compact structure of receptors.

### Hydrogen Bonding

**Figure 8:**
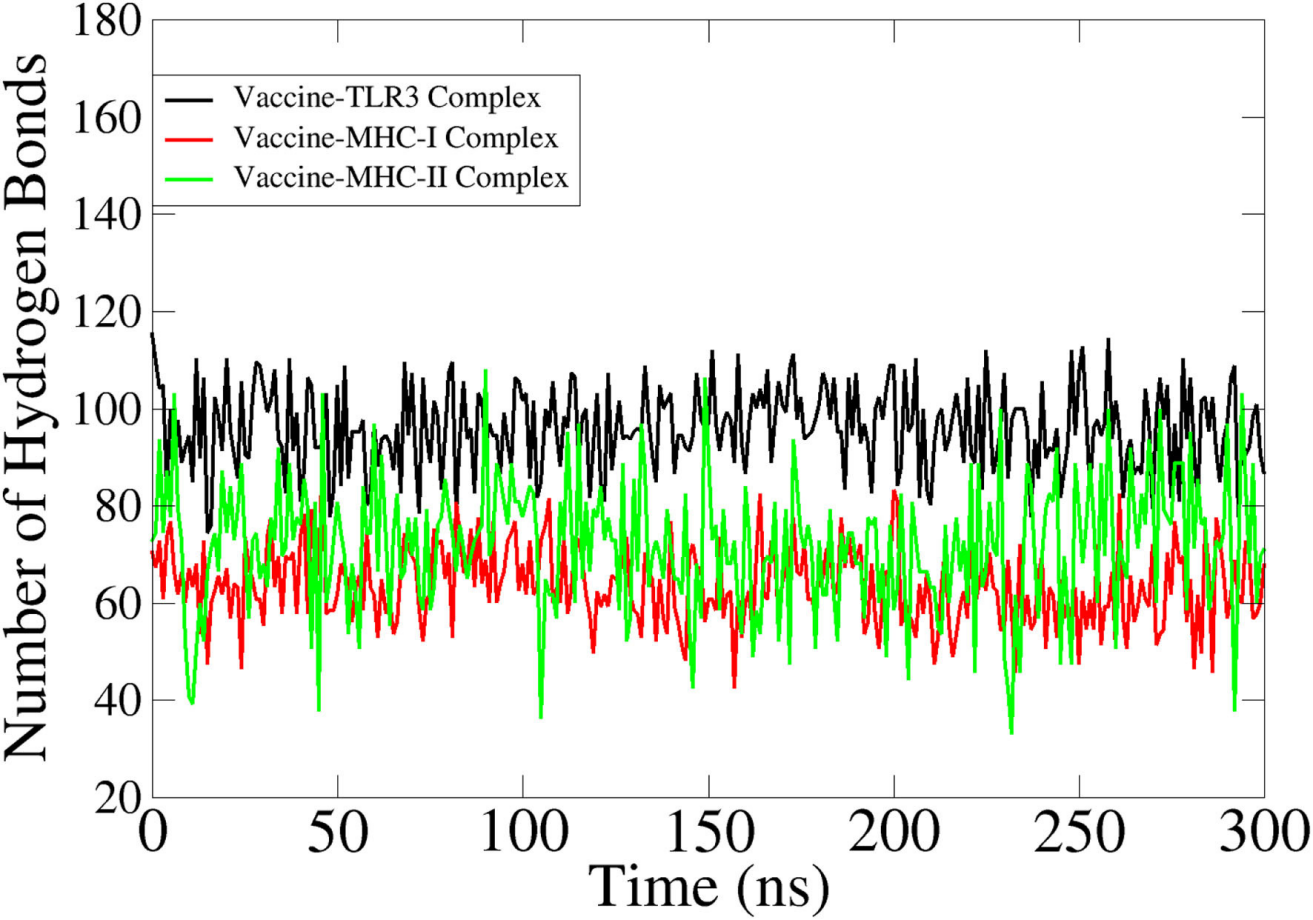
Hydrogen bonds analysis between the vaccine and innate immune receptors.

Hydrogen bonds play a critical role in determining the specificity of molecular recognition and hold vital importance in vaccine-receptors interactions (Hubbard and Kamran Haider, 2001). The number of hydrogen bonds in each simulation frame was determined to investigate the intermolecular strength of binding between vaccine and receptors. The vaccine on average produced 100 hydrogen bonds with TLR3, 86 with MHC-I, and 82 with MHC-II. Figure 8 depicts the number of hydrogen bonds in all 3 systems.

### Intermolecular Binding Free Energies

The binding free energies of vaccine-receptor complexes were estimated using the MMPB\GBSA method (Genheden and Ryde, 2015). The net energy value of systems was very low, indicating high interaction affinity between binding molecules. In MMGBSA, the net binding value for TLR3 vaccine is −166.41 kcal/mol, whereas, in MMPBSA, it is −172.04 kcal/mol (Table 4). In MMGBSA and MMPBSA, the net energy value of MHC-I vaccine is −230.05 and −224.72 kcal/mol, respectively, while for MHC-II vaccine, the binding free energy is −195.2 kcal/mol (MMGBSA) and −203.24 (MMPBSA). The gas phase energy is very dominant and contributes considerably to the total binding energy in all systems. In both MMGBSA and MMPBSA, TLR3 vaccine has gas-phase energy of −199.34 kcal/mol (the van der Waals contribution to the net gas phase energy is −120.17 kcal/mol, and electrostatic energy is −79.17 kcal/mol). For TLR3 vaccine, solvation energy contributes 32.93 kcal/mol to the MMGBSA and 27.3 kcal/mol in MMPBSA, whereas MH-I vaccine gas-phase energy is −262.34 kcal/mol (the van der Waals contribution is −166.87 kcal/mol, and electrostatic energy is −95.47 kcal/mol). Solvation energy contributes 32.29 kcal/mol in MMGBSA and 37.62 kcal/mol in MMPBSA. The net MMPBSA for MHC-II vaccine is higher than the MMGBSA. The contributions of gas phase to the system net energy are significantly higher (−233.39 kcal/mol) with van der Waals and electrostatic contribution being −160.78 and −72.61 kcal/mol, respectively.

**Table 4:** Estimation of binding free energies for complexes.

**Energy parameter**	**TLR-3-Vaccine complex**	**MHC-I-Vaccine complex**	**MHC-II-Vaccine complex**
**MM-GBSA**			
Van der Waals energy	−120.17	−166.87	−160.78
Electrostatic energy	−79.17	−95.47	−72.61
Polar solvation energy	41.00	42.84	48.19
Non-polar solvation energy	−8.07	−10.55	−10.00
Gas phase energy	−199.34	−262.34	−233.39
Solvation energy	32.93	32.29	38.19
Total	−166.41	−230.05	−195.2
**MM-PBSA**			
Van der Waals energy	−120.17	−166.87	−160.78
Electrostatic energy	−79.17	−95.47	−72.61
Polar solvation energy	39.47	46.87	38.52
Non-polar solvation energy	−12.17	−9.25	−8.37
Gas phase energy	−199.34	−262.34	−233.39
Solvation energy	27.3	37.62	30.15
Total	−172.04	−224.72	−203.24

### Disulfide Engineering of Vaccine

**Figure 9:**
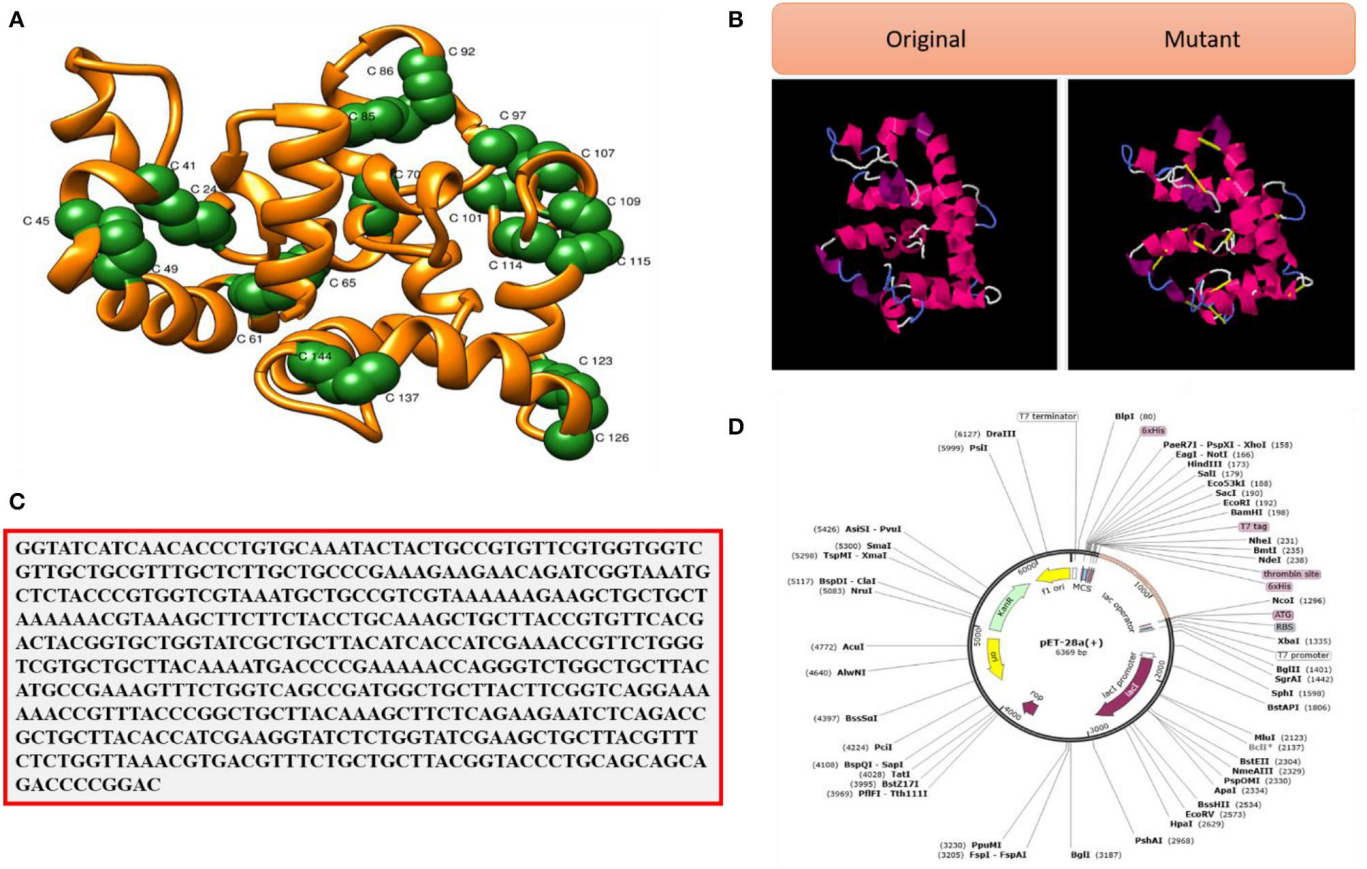
**(A)** Pair of residues (shown as green balls) having high unfavorable energy and were mutated. **(B)** Disulfide engineering (original vs. mutated structures). **(C)** Codon optimized sequence and **(D)** Cloned VACCINE (shown in violet color) into expression vectors.

Strengthening of the vaccine structure is critical for a variety of biotechnological applications (Dombkowski et al., 2014). Figure 9A illustrates the disulfide engineered vaccine model. Thirteen pairs of residues were chosen for disulfide engineering. These residues are Gly1-Asn4, Cys24-Cys41, Lys45-Ala49, Ala61-His65, Gly70-Gly82, Leu81-Ala85, Tyr86-Asn92, Ala97-Met107, Lys101-Gly104, Ala108-Glu114, Ala109-Lys115, Lys123-Gln126, and Glu137-Ala144. The average Chi3 and energy values for the said residue pairs are −31.80 (max, 113.17 and min, −105.35) and 3.90, respectively (maximum, 6.07 kcal/mol and minimum, 1.43 kcal/mol). Figure 9B represents the original vs. mutant vaccine ensemble.

### Optimization of Codons and *in silico* Cloning

Maximum vaccine expression is particularly desired in experimental research, which depends on the codon optimization of the given amino acid sequence as per host expression system. The CAI and GC content of the vaccine is in the ideal range; CAI value, 1.0, and GC contents, 50.0% (Abbas et al., 2020). These data suggest good vaccine expression in the *E. coli* K12 strain. The improved vaccine sequence is given in Figure 9C, while the cloned vaccine sequence is presented in Figure 9D. The vaccine at the ends is tagged with 6 × histidine to facilitate purification of the vaccine molecule.

## Conclusion

In this study, existing immunoinformatic methodologies were employed to rank potential vaccine candidates against MCV. B- and T-cells epitopes that fulfilled all the vaccine design parameters were selected for multiepitope vaccine designing. The vaccine generates strong immune responses, as well as interferons and cytokines. The docking analysis interpreted the vaccine to show strong binding with different innate immune receptors and is dynamically stable. The atomic level binding free energies estimation also validated the docking and simulation findings. Despite the need for further experimental testing in suitable animal models to determine the true efficacy of the designed vaccine, the computational predictions might provide early epitopes for vaccine development and to treat and prevent MCV.

## Data Availability Statement

The original contributions presented in the study are included in the article/Supplementary Material, further inquiries can be directed to the corresponding author/s.

## Author Contributions

NA designed, performed, and wrote this study.

## Conflict of Interest

The author declares that the research was conducted in the absence of any commercial or financial relationships that could be construed as a potential conflict of interest.

## Publisher's Note

All claims expressed in this article are solely those of the authors and do not necessarily represent those of their affiliated organizations, or those of the publisher, the editors and the reviewers. Any product that may be evaluated in this article, or claim that may be made by its manufacturer, is not guaranteed or endorsed by the publisher.
