# Supplementary material for: Immunoinformatics- and Bioinformatics-Assisted Computational Designing of a Novel Multiepitopes Vaccine Against Cancer-Causing Merkel Cell Polyomavirus
Source: Front Microbiol. 2022 Jun 28;13:929669. doi: 10.3389/fmicb.2022.929669 (PMC9273964; doi:10.3389/fmicb.2022.929669)
Supplement: Supplementary file 1 [file Data_Sheet_1.docx]

**Table S1.** Top-20 solutions generated by patch dock in docking of vaccine against each innate immune receptor.

| **TLR3** | | | | |
| --- | --- | --- | --- | --- |
| **Solution No** | **Score** | **Area** | **Atomic contact energy** | **Transformation** |
| 1 | 16800 | 2410 | 420.52 | -3.11 -0.95 1.54 -85.43 -15.98 111.81 |
| 2 | 16370 | 2586.5 | 388.63 | -1.07 -1.40 2.09 37.48 23.67 75.13 |
| 3 | 15972 | 2366.9 | 473.37 | 2.44 -1.02 1.05 -112.10 -13.07 78.50 |
| 4 | 15768 | 2252.7 | 499.70 | -0.50 -0.78 -2.53 -22.07 72.60 50.78 |
| 5 | 15756 | 1926.8 | 339.04 | -1.18 -0.61 3.10 10.58 46.18 81.99 |
| 6 | 15692 | 2104 | 498.83 | -2.78 0.28 -1.61 5.34 41.83 65.46 |
| 7 | 15436 | 2127.4 | 494.67 | -2.16 0.80 0.27 -120.60 -66.95 26.42 |
| 8 | 15324 | 2171 | 496.40 | 2.70 0.40 -1.06 13.47 57.32 22.91 |
| 9 | 15128 | 2363.4 | 370.88 | -2.69 0.24 -1.49 -9.10 42.52 75.13 |
| 10 | 14848 | 2072.4 | 410.80 | -0.23 1.45 -3.10 -59.81 42.59 -48.97 |
| 11 | 14814 | 2356.8 | 364.88 | 1.91 -0.39 -1.89 60.57 6.33 16.59 |
| 12 | 14812 | 1879 | 233.31 | 1.31 -1.40 0.19 -114.61 -7.16 66.14 |
| 13 | 14600 | 1876.6 | 435.44 | 2.84 0.86 -1.97 85.15 -2.52 0.73 |
| 14 | 14588 | 1802.9 | 467.93 | -0.32 1.14 -1.35 -98.95 -50.37 -57.72 |
| 15 | 14502 | 1856 | 430.21 | 2.83 0.62 -1.14 15.42 56.86 14.72 |
| 16 | 14424 | 2059.4 | 423.07 | 2.04 -1.05 -1.52 48.92 31.82 59.26 |
| 17 | 14264 | 1729.8 | 371.35 | 0.79 -0.86 1.54 -28.93 -122.55 17.70 |
| 18 | 14108 | 1989.1 | 376.75 | 1.51 0.37 -0.72 -13.75 32.32 -54.67 |
| 19 | 14028 | 1716.3 | 360.40 | -1.29 0.49 2.62 79.10 -3.50 28.16 |
| 20 | 14014 | 1652.9 | 317.60 | 2.56 0.57 -1.15 29.51 48.64 -3.22 |
| **MHC-I** | | | | |
| **Solution No** | **Score** | **Area** | **Atomic contact energy** | **Transformation** |
| 1 | 15472 | 2911.7 | 202.07 | -2.29 -1.21 2.58 -21.23 27.97 64.87 |
| 2 | 14310 | 1909.6 | 345.77 | -3.05 0.04 -0.02 -68.37 52.05 33.20 |
| 3 | 14148 | 2249.8 | 372.95 | 2.83 0.31 2.35 16.66 -99.02 -5.45 |
| 4 | 14142 | 1916.4 | 399.65 | 0.46 -1.23 -1.04 -48.09 63.75 7.99 |
| 5 | 14120 | 1783.1 | 494.79 | 2.67 -0.56 2.29 -34.91 -85.71 21.81 |
| 6 | 13974 | 1986.4 | 472.11 | -2.99 0.90 2.21 36.03 -100.40 -28.40 |
| 7 | 13812 | 1730.2 | 498.90 | -1.33 -0.30 3.08 26.41 76.22 49.82 |
| 8 | 13770 | 1859 | 448.72 | -0.19 -0.97 2.63 81.09 22.02 5.23 |
| 9 | 13746 | 1615.1 | 421.48 | 2.41 1.08 0.13 -47.83 71.41 -76.19 |
| 10 | 13592 | 2001.5 | 282.94 | -1.98 0.46 -0.48 -74.91 13.11 11.70 |
| 11 | 13462 | 1754.8 | 378.73 | -1.62 -0.25 0.20 -8.73 -66.84 52.29 |
| 12 | 13260 | 1675.9 | 90.44 | -0.45 -0.09 -3.06 40.83 64.35 -16.18 |
| 13 | 13234 | 1996.1 | 341.28 | 2.36 0.06 -0.90 43.37 95.72 -31.99 |
| 14 | 13232 | 2508.7 | 334.60 | -2.74 0.16 -1.45 35.67 70.66 40.62 |
| 15 | 13194 | 2226.2 | 223.40 | -2.22 -1.49 -0.11 58.76 -17.47 49.41 |
| 16 | 13186 | 2221.4 | 383.95 | -1.17 0.59 -1.22 -70.16 34.79 -31.16 |
| 17 | 13134 | 1710.6 | 299.69 | -2.38 -0.74 -0.65 -3.92 -0.67 75.71 |
| 18 | 13084 | 1963.9 | -17.09 | 0.55 0.87 -2.56 -10.23 0.50 -121.49 |
| 19 | 13026 | 1776.1 | 235.45 | -0.48 -1.10 2.04 83.24 -10.44 17.55 |
| 20 | 13006 | 2315.6 | 324.14 | -1.31 0.66 -0.43 -70.60 -38.21 -24.90 |
| **MHC-II** | | | | |
| **Solution No** | **Score** | **Area** | **Atomic contact energy** | **Transformation** |
| 1 | 14404 | 2044.7 | 292.42 | 1.64 0.23 -0.56 4.93 53.13 -56.15 |
| 2 | 14268 | 1875.8 | 93.68 | -1.69 0.48 1.98 97.04 -53.65 17.66 |
| 3 | 13800 | 2068 | 95.58 | -1.10 0.90 -1.68 -64.37 45.79 -13.17 |
| 4 | 13784 | 1752.6 | 249.03 | -2.88 0.47 0.62 -119.17 -9.68 31.63 |
| 5 | 13588 | 2208.6 | 319.70 | -0.94 -0.43 -0.08 -40.13 -62.70 42.68 |
| 6 | 13564 | 1555.4 | 373.90 | -2.32 -0.22 -0.55 -14.10 15.02 80.47 |
| 7 | 13422 | 1719 | 381.91 | -0.99 -0.30 2.53 58.80 51.22 34.56 |
| 8 | 13348 | 1824.4 | 347.81 | -2.92 -0.97 -0.27 3.54 31.68 81.90 |
| 9 | 13334 | 1686.3 | 275.62 | -1.08 -0.30 -0.99 -74.10 0.92 23.84 |
| 10 | 13114 | 2226.8 | 120.85 | 0.17 0.17 -0.84 -67.90 -22.11 -58.43 |
| 11 | 13074 | 1802.1 | 421.83 | -0.15 0.87 2.39 64.75 58.08 -73.69 |
| 12 | 13064 | 2003.6 | 181.56 | -2.75 -0.07 1.39 -21.87 -42.88 75.13 |
| 13 | 13058 | 2130.9 | 171.22 | -3.10 -0.20 -1.33 44.74 55.00 41.80 |
| 14 | 13046 | 1756.5 | 294.72 | -1.58 0.61 -0.84 -78.52 33.52 9.15 |
| 15 | 13038 | 1640.7 | 332.17 | 2.60 0.73 1.28 -88.76 -5.88 -20.61 |
| 16 | 12970 | 1668 | 181.60 | 0.29 -1.37 -2.62 20.42 72.13 43.17 |
| 17 | 12946 | 1913.1 | -50.20 | -1.22 1.30 -2.43 -0.73 76.10 -27.04 |
| 18 | 12898 | 2017.5 | 374.99 | 0.64 -1.17 -2.60 46.88 60.73 21.99 |
| 19 | 12848 | 1677.6 | 82.54 | 1.21 0.76 0.22 4.78 11.46 -76.78 |
| 20 | 12848 | 1907.6 | 465.18 | 1.49 0.69 -0.53 34.39 31.54 -69.10 |

**Table S2.** FireDock ranking of docked solutions. The solution ranked on top was considered for further analysis.

| **TLR3-Construct** | | | | | | |
| --- | --- | --- | --- | --- | --- | --- |
| **Rank** | **Solution Number** | **Global Energy** | **Attractive van der Waals** | **Repulsive van der Waals** | **Atomic contact energy** | **Hydrogen bond energy** |
|  |  | ↓ |  |  |  |  |
| 1 | 7 | -23.22 | -20.40 | 8.70 | 4.08 | -4.81 |
| 2 | 9 | 7.87 | -11.42 | 5.81 | 6.34 | -1.32 |
| 3 | 10 | 11.08 | -35.27 | 16.43 | 17.25 | -3.77 |
| 4 | 6 | 11.92 | -30.68 | 17.47 | 20.77 | -5.81 |
| 5 | 3 | 20.36 | -3.95 | 0.00 | 6.84 | 0.00 |
| 6 | 1 | 20.97 | -10.35 | 60.79 | -2.42 | -0.98 |
| 7 | 8 | 23.16 | -8.33 | 5.75 | 8.73 | -1.93 |
| 8 | 5 | 31.40 | -11.06 | 19.06 | 8.77 | -2.72 |
| 9 | 2 | 196.54 | -26.10 | 260.86 | 14.56 | -1.60 |
| 10 | 4 | 1142.16 | -16.23 | 1427.97 | 4.00 | -4.26 |
| **MHC-I Vaccine** | | | | | | |
| **Rank** | **Solution Number** | **Global Energy** | **Attractive van der Waals** | **Repulsive van der Waals** | **Atomic contact energy** | **Hydrogen bond energy** |
|  |  | ↓ |  |  |  |  |
| 1 | 5 | -5.82 | -26.69 | 8.72 | 12.17 | -1.84 |
| 2 | 2 | -4.30 | -22.36 | 7.30 | 9.37 | -3.16 |
| 3 | 1 | -3.90 | -15.53 | 11.12 | 9.80 | -1.53 |
| 4 | 9 | 14.52 | -37.43 | 31.72 | 15.88 | -5.16 |
| 5 | 4 | 15.93 | -33.47 | 9.16 | 11.56 | -3.79 |
| 6 | 10 | 16.19 | -36.26 | 37.86 | 14.66 | -4.10 |
| 7 | 3 | 21.38 | -26.15 | 57.33 | 7.81 | -4.23 |
| 8 | 8 | 22.76 | -32.25 | 45.05 | 20.02 | -7.29 |
| 9 | 6 | 25.91 | -33.74 | 30.28 | 22.68 | -5.74 |
| 10 | 7 | 27.46 | -27.12 | 13.02 | 17.94 | -7.84 |
| **MHC-II Vaccine** | | | | | | |
| **Rank** | **Solution Number** | **Global Energy** | **Attractive van der Waals** | **Repulsive van der Waals** | **Atomic contact energy** | **Hydrogen bond energy** |
|  |  | ↓ |  |  |  |  |
| 1 | 2 | -7.91 | -17.34 | 10.66 | 3.54 | -1.77 |
| 2 | 4 | 0.20 | -10.91 | 18.52 | -0.58 | -1.37 |
| 3 | 8 | 1.37 | -26.78 | 23.73 | 11.76 | -1.47 |
| 4 | 5 | 2.95 | -4.90 | 1.44 | 0.17 | -0.76 |
| 5 | 6 | 7.20 | -37.59 | 10.01 | 20.19 | -3.82 |
| 6 | 3 | 9.83 | -4.27 | 2.62 | 2.83 | 0.00 |
| 7 | 1 | 10.94 | -3.70 | 0.00 | 3.46 | -0.96 |
| 8 | 7 | 21.98 | -6.13 | 0.59 | 4.28 | -1.24 |
| 9 | 10 | 29.47 | -4.74 | 0.00 | 3.59 | 0.00 |
| 10 | 9 | 41.95 | -37.90 | 121.19 | 15.16 | -2.37 |
